# Microcollinearity in an ethylene receptor coding gene region of the *Coffea canephora *genome is extensively conserved with *Vitis vinifera *and other distant dicotyledonous sequenced genomes

**DOI:** 10.1186/1471-2229-9-22

**Published:** 2009-02-25

**Authors:** Romain Guyot, Marion de la Mare, Véronique Viader, Perla Hamon, Olivier Coriton, José Bustamante-Porras, Valérie Poncet, Claudine Campa, Serge Hamon, Alexandre de Kochko

**Affiliations:** 1 UMR GDP, IRD BP 64501, Centre IRD de Montpellier, BP 64501, Montpellier Cedex 5, France; 2 UMR DIA-PC, IRD Génomique Comparative et Fonctionnelle de l'Adaptation, Centre IRD de Montpellier, BP 64501, Montpellier Cedex 5, France; 3 UMR 118, INRA Agrocampus Rennes Amélioration des Plantes, Domaine de la Motte – BP 35327, 35650 Le Rheu cedex, France

## Abstract

**Background:**

*Coffea canephora*, also called Robusta, belongs to the Rubiaceae, the fourth largest angiosperm family. This diploid species (2x = 2n = 22) has a fairly small genome size of ≈ 690 Mb and despite its extreme economic importance, particularly for developing countries, knowledge on the genome composition, structure and evolution remain very limited. Here, we report the 160 kb of the first *C. canephora *Bacterial Artificial Chromosome (BAC) clone ever sequenced and its fine analysis.

**Results:**

This clone contains the *CcEIN4 *gene, encoding an ethylene receptor, and twenty other predicted genes showing a high gene density of one gene per 7.8 kb. Most of them display perfect matches with *C. canephora *expressed sequence tags or show transcriptional activities through PCR amplifications on cDNA libraries. Twenty-three transposable elements, mainly Class II transposon derivatives, were identified at this locus. Most of these Class II elements are Miniature Inverted-repeat Transposable Elements (MITE) known to be closely associated with plant genes. This BAC composition gives a pattern similar to those found in gene rich regions of *Solanum lycopersicum *and *Medicago truncatula *genomes indicating that the *CcEIN4 *regions may belong to a gene rich region in the *C. canephora *genome. Comparative sequence analysis indicated an extensive conservation between *C. canephora *and most of the reference dicotyledonous genomes studied in this work, such as tomato (*S. lycopersicum*), grapevine (*V. vinifera*), barrel medic *M. truncatula*, black cottonwood (*Populus trichocarpa*) and *Arabidopsis thaliana*. The higher degree of microcollinearity was found between *C. canephora *and *V. vinifera*, which belong respectively to the Asterids and Rosids, two clades that diverged more than 114 million years ago.

**Conclusion:**

This study provides a first glimpse of *C. canephora *genome composition and evolution. Our data revealed a remarkable conservation of the microcollinearity between *C. canephora *and *V. vinifera *and a high conservation with other distant dicotyledonous reference genomes. Altogether, these results provide valuable information to identify candidate genes in *C. canephora *genome and serve as a foundation to establish strategies for whole genome sequencing. Future large-scale sequence comparison between *C. canephora *and reference sequenced genomes will help in understanding the evolutionary history of dicotyledonous plants.

## Background

Twenty years ago, the availability of several plant genetic maps allowed the development of the first comparative genetic mapping studies [1]. Results indicated that the marker content and order were globally conserved within plant families, between species that sometimes display huge differences in genome size and organization. Thus, macrosynteny was established within the Brassicaceae, the *Fabaceae*, the *Solanaceae *and the *Poaceae *families [2]. In *Poaceae*, the spectacular conservation of macrosynteny gave the opportunity to draw a consensus map for seven different grass genomes that diverged more than 50 million years ago [3]. In contrast to within family comparative mapping, macrosynteny appears less conserved between distantly related species belonging to distant families or different clades [4,5]. With such a comparison, the overall conservation of the genome organization appears scrambled by mechanisms of genome evolution such as chromosome rearrangements and genome duplications, to such an extent that the synteny was frequently limited over small genetic intervals. Recently, the increase of complete or partial genomic sequence of reference plant species from different families and the development of the BAC clones sequencing in experimental species gave the opportunity to perform direct comparisons at the genomic sequence level. Beside intra-family comparisons, microcollinearity was investigated between Arabidopsis and tomato [6,7], Arabidopsis, tomato and *Capsella *[8], Arabidopsis, *M. truncatula*, *P. trichocarpa *and *Lotus japonicus *[5] and Arabidopsis, Medicago, Populus and *Cucumis melo *[9]. Despite a lack of macrosynteny, a significant microcollinearity was established between these phylogenetically distant species. In addition, direct sequences comparisons allowed the detection of a complex network of microcollinearity between Arabidospis and tomato [7] and between Arabidospsis, Medicago and Populus [5] suggesting the presence of ancient segmental duplication in the Arabidopsis genome. Altogether, these analyses provided new insight into the plant genome structure and evolution.

The *Rubiaceae *family (Euasterids I clade) is one of the largest angiosperm families, comprising about 600 genus and more than 13,000 species and including the *Gardenia*, the *Cinchona *and the *Coffea *genus. The genus *Coffea *comprises two economically important crop species: *C. arabica *L. and *C. canephora *Pierre that account respectively for 65% and 35% of the worldwide coffee production (International Coffee Organization, ). *C. canephora *represents a model genome in the *Rubiaceae *family as it has a diploid genome (2n = 2x = 22), a relatively small genome size (2C = 1.43 pg) [10] compared to the allotetraploid genome of *C. arabica *and an extensive genetic diversity [11]. Moreover, genomic resources have been recently established for *C. canephora*, such as a BAC library [12] and the availability of 55,840 *C. canephora *ESTs from different tissues and stages of seed development [13] to facilitate the isolation and the characterization of genes with agronomic interest. However, despite its agronomical importance, very little information concerning the composition, the structure and the evolution of the *C. canephora *genome is now available. Particularly, it remains unclear so far how the distantly related sequenced dicotyledonous reference genomes such as Arabidopsis, Medicago, Populus, grapevine and tomato will have a predictive value to study the genome structure and to isolate genes of interest in *C. canephora*. Recently, in an effort to identify factors implicated in ethylene perception during the ripening of the *C. canephora *berries, several ethylene receptors and transcription factors genes were isolated [14-16]. One of them called *CcEIN4 *was identified on an isolated BAC clone [15].

In this study, we present the complete sequencing of the *C. canephora *BAC clone 46C02, carrying the *CcEIN4 *gene. Sequence analysis of the 160 kb of the BAC indicates a high density of active genes and few transposable elements. Sequence comparisons reveal a high microcollinearity between the *C. canephora *BAC clone and most of the current dicotyledonous sequenced genomes that diverged 114 to 125 Million Years Ago (MYA).

## Results

### The *C. canephora *BAC clone 46C02 carries the mono locus *CcEIN4 *gene

The *CcEIN4 *gene, used as a probe for hybridization, revealed eight positive BAC clones on the high-density filters of the *C. canephora *BAC library. Among these clones, the BAC clone 46C02 was randomly chosen for further analyses. Low-pass sequencing of about 70 sub-clones from BAC 46C02 suggested the presence of several potential coding regions and sequence analyses confirmed the presence of the *CcEIN4 *gene. Finally, pulsed field analysis indicated the presence of a large insert of about 160 kb length.

**Figure 1:**
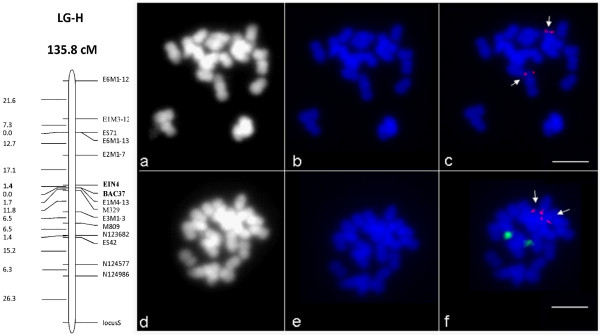
**Genetic and physical mapping of BAC 46C02**. **A. Genetic mapping of markers located onto the BAC 46C02**. Linkage Group H (LG H) from the [(*C. canephora *× *C. heterocalyx*) × *C. canephora*] genetic map, showing the location of the EIN4 gene and BAC-37 microsatellites. B. Physical mapping by BAC-FISH on mitotic chromosomes from . (a-c) Fluorescence in situ hybridization (FISH) signals of the BAC clone 46C02 (red signals) on mitotic metaphase chromosomes of *C. canephora *(genotype BB 62). Fluorescence signals of the BAC clone are indicated with arrows. (d-f) Double FISH with the BAC clone 46C02 (red signals) and rDNA (green signals). Chromosomes were counterstained with DAPI (blue). Bars represent 5 μm. (a and d), grayscale images of the same preparation as in b and e.

Segregation analysis of the EIN4 marker, in the interspecific back-cross progeny [(*C. canephora *× *C. heterocalyx*) × *C. canephora*], showed mono locus Mendelian segregation pattern (Figure 1A). In addition, *in situ *BAC hybridizations (BAC-FISH) showed a unique and chromosome-specific sub-terminal/terminal labeling for 46C02 in a non-45S rDNA chromosome pair (Figure 1B).

### Sequence composition and organization of BAC 46C02

#### Genes

The *C. canephora *BAC, containing the *C*c*EIN4 *gene, was fully sequenced. The 160,404 bp of the BAC clone 46C02 (GenBank accession EU164537) showed an overall 37.3% GC content and a GC content of the predicted coding sequences (CDS) of 45.7%.

*Ab initio *predictions gave 30, 43 and 55 predicted gene structures with the FGENESH gene finder trained respectively for grapevine, tomato and tobacco gene models. We evaluated the accuracy of the predictions by comparative analysis between predicted gene structures and results of similarity searches of the BAC sequence against proteins and nucleotides sequences databases. Manual controls between *ab initio *predictions and sequences alignments suggest numerous incongruities. Over prediction of inaccurate gene models, imprecise exons, mainly located at the 3' ends of predicted genes supported by EST alignments and inexact predictions of exon-intron boundaries (start, stop and splicing sites) was observed. These results indicate a relatively low quality of the *ab initio *predictions in *C. canephora *when using training set of gene models from different eudicots model genomes. In absence of available *C. canephora *training set, we conclude that prediction of the gene structures may be used cautiously.

**Figure 2:**
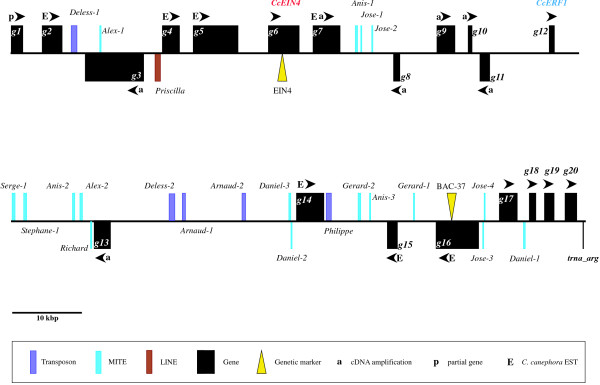
**Physical map of the 160,404 bp sequence of the coffee BAC 46C02**. Black boxes represent identified coding regions and arrowheads indicate transcriptional orientation of genes. The *CcEIN4 *gene is indicated in red. Colored boxes represent identified transposable elements as follows: violet for transposons, blue for MITEs and brown for LINE. Markers used for genetic mapping on LGH (EIN4 and BAC-37) are indicated by a yellow triangle. P indicates partial gene, whereas a and E symbolize respectively successful gene amplification on *C. canephora *cDNA libraries and strong identities with *C. canephora *coffee ESTs.

In total, 21 genes were identified and validated by sequence alignments, giving an overall gene density of about one gene per 7.8 kb, considering the partial gene (*g1*) that covers the 5' part of the BAC insert (Table 1; Figure 2). Similarities with plant Expressed Sequence Tag sequences (EST) were found for the 21 identified genes (Table 1). Eight genes (*g2, g4, g5, g6, g7, g14, g15 *and *g16*) have almost perfect matches with *C. canephora *ESTs and mRNA, with sequence identities higher than 97%, suggesting that these genes are expressed. All the remaining genes have significant matches with plant ESTs (> 70% identity). On the 10 genes analyzed, seven (*g3, g7, g8, g9, g10, g11 *and *g13*) showed PCR amplifications on two *C. canephora *cDNA libraries (Table 1). For genes *g3 *and *g7*, sequencing of PCR products allowed the fine determination of the gene model and then the re-annotation of the gene predictions. Gene 6 (*g6*) encodes an ethylene receptor with a high sequence identity (85.7%) with the tomato ethylene receptor neverripe gene (*ETR5 *accession AY600439, [17]). This gene, called *CcEIN4 *(position from 36174 to 40482 bp), was previously cloned and analyzed in our lab [15]. On the 19 remaining coding genes, 16 contain known protein domains in pfam database [18] (Table 1). Among them, a gene coding for an ERF/AP2 transcription factor has been identified from 75,661 to 76,365 bp (*g12*, *CcERF1*).

**Table 1:** List of identified genes in the *C. canephora *BAC 46C02.

**Gene Name**	**Product**	**Protein domain**	**Best BLASTN homology**	**Best BLASTX homology**	**Best BLASTN EST homology**
**46C02_g1^pb^**	Putative protein	pfam01843, DIL, DIL domain	NM_001064205 *O. sativa *(1e-148)	CAO70643 unnamed protein product *V. vinifera *(3e-119)	AJ796769 *A. majus *(0.0)
**46C02_g2**	Expressed Protein	pfam05764, YL1 nuclear protein	NM_129229 *A. thaliana *(5e-80)	NP_181212 DNA binding A. thaliana (1e-90)	**DV701332 *C. canephora *(0.0; 99% id.)**
**46C02_g3^a^**	Putative protein	pfam02791, DDT domain	NM_117344 *A. thaliana *(9e-83)	CAO47883 *V. vinifera *(0.0)	DY269367 *C. clementina *(9e-133)
**46C02_g4**	Expressed protein	/	EF147735 *P. trichocarpa *(2e-52)	ABL97988 putative c-myc *B. rapa *(4e-35)	**DV692405 *C. canephora *(2e-167; 100% id.)**
**46C02_g5**	Expressed protein	pfam03479, DUF296, Domain of unknown function	AJ132349 A. majus (3e-96)	CAO65023 unnamed protein product *V. vinifera *(4e-65)	**DV692183 *C. canephora *(0.0; 97% id.)**
**46C02_g6^c^**	***CcEIN4***	cd00156, REC, Signal receiver domain	AF118844 *L. esculentum *(0.0)	AF118844 ethylene receptor *L. esculentum *(0.0)	CK272769 *S. tuberosum *(0.0)
**46C02_g7^a^**	Expressed protein	pfam08242, Methyltransferase domain	BT014095 *L. esculentum *(8e-64)	CAO65026 unnamed protein product *V. vinifera *(2e-113)	**DV685577 *C. canephora *(0.0; 99% id.)**
**46C02_g8^a^**	Putative protein	pfam03479, DUF296, Domain of unknown function	AM463589 *V. vinifera *(3E-158)	CAO65027 unnamed protein product *V. vinifera *(8e-83)	EB084622 *C. annuum *(3e-150)
**46C02_g9^a^**	Putative protein	/	NM_111327 *A. thaliana *(7e-123)	CAO65029 unnamed protein product *V. vinifera *(5e-122)	BI925858 *S. lycopersicum *(3e-114)
**46C02_g10^a^**	Putative protein	cd01926, cyclophilin_ABH_like	AK246441 *S. lycopersicum *(2E-112)	CAN61038 hypothetical protein *V. vinifera *(7e-70)	N980378 *S. chacoense *(1e-114)
**46C02_g11^a^**	Putative protein	/	AM448871 *V. vinifera *(5E-75)	CAO65032 unnamed protein product *V. vinifera *(1e-112)	CK272594 *S. tuberosum *(2e-79)
**46C02_g12^b^**	*CcERF1*	smart00380, AP2 DNA-binding domain	AM441538 *V. vinifera *(7E-38)	CAO65033 unnamed protein product *V. vinifera *(3e-53)	CV262586 *P. trichocarpa *(3e-36)
**46C02_g13^a^**	Putative protein	pfam00082, Peptidase_S8; cd02120, subtilisin_like	AP009276 *S. lycopersicum *(8e-170)	CAA06414 P69F protein *S. lycopersicum *(0.0)	CK269227 *S. tuberosum *(5e-108)
**46C02_g14**	Expressed protein	smart00156, PP2Ac, Protein phosphatase 2A	AJ002485 *M. sativa *(0.0)	BAF31132 subunit of protein phosphatase 1 *V. faba *(2e-168)	**DV705485 *C. canephora *(0.0; 100% id.)**
**46C02_g15**	Expressed protein	cd00180, Serine/Threonine protein kinase	AF453448 *S. tuberosum *(1e-161)	AF203481 carboxylase kinase *L. esculentum *(2e-126)	**DV693205 *C. canephora *(0.0; 99% id.)**
**46C02_g16**	Expressed protein	pfam01501, Glycosyl transferase family 8	BT013608 *L. esculentum *(0.0)	CAO65058 unnamed protein product *V. vinifera *(0.0)	**DV705148 *C. canephora *(0.0; 99% id.)**
**46C02_g17^b^**	Putative protein	pfam00067, Cytochrome P450	CU104691 *S. lycopersicum *(2e-30)	CAO66223 unnamed protein product *V. vinifera *(2e-109)	CF513973 *V. vinifera *(1e-37)
**46C02_g18**	Putative protein	pfam07859, Abhydrolase_3	AC209222.1 *P. trichocarpa *(1e-23)	CAO47785 unnamed protein product *V. vinifera *(1e-46)	DV677672 *C. canephora *(8e-94; 80% id.)
**46C02_g19**	Putative protein	cd00167 DNA-binding domains	EU181424 *V. vinifera *(6e-84)	AAB41101 transcription factor Myb1 *N. tabacum *(7e-60)	FC069164 *V. vinifera *(5e-84)
**46C02_g20^b^**	Putative protein	Pfam00190, Cupin_1	X82463 *M. salicifolia *(9e-27)	CAA57846 *M. salicifolia *(4e-82)	EE986213 *N. nucifera *(1e-23)
**46C02_g21**	tRNA Arg	/	CU104691 *S. lycopersicum *(9e-29)	/	EX530859 *M. truncatula *(5e-25)

^a^detected by cDNA amplification, ^b ^not detected by cDNA amplification, ^p ^Partial gene, ^c ^Bustamante-Porras et al., 2006

Finally, an unequal distribution of genes was observed along the BAC sequence. The identified genes were distributed in two main locations (from 1 to 76,365 bp and from 119,888 to 160,243 bp, Figure 2) with respectively twelve (g1 – g12) and seven genes (g14 – g20) in the 5' proximal and 3' distal parts of the BAC. The g1–g12 group of gene covers ~76 kb with a gene density of approximately one gene per 6.3 kb whereas the g14–g20 group covers a distance of 40.5 kb with a gene density of one gene per 5.7 kb. The central part of the BAC, containing only one gene (*g13*), covers a distance of 43 kb (Figure 2).

#### Repeated sequences

##### Simple sequence repeats (SSRs)

The availability of the first *C. canephora *BAC sequence allowed us to identify the SSR density in genomic sequences. There were 39 SSRs along the 160,404 bp of the BAC 46C02, giving an overall genomic SSR density of one SSR every 4.1 kb. Most of them (32 SSRs) were simple repeat motifs. The remaining SSRs were di-nucleotides [CT]_20_, [CT]_9_, [TC]_9_, [AT]_9 _and [TA]_18 _and tri-nucleotidesmotifs [GAA]_6 _and [ATG]_7_. One of the di-nucleotides SSRs [TC]_9_, called BAC-37, found in the fourth intron of the gene *g16*, coding for a putative Glycosyl transferase, was mapped on the same linkage group H of the [(*C. canephora *× *C. heterocalyx*) × *C. canephora*] genetic map as the EIN4 marker (Figure 1A and 2). The two markers were 1.4 cM apart.

##### Transposable elements (TEs)

In addition to Simple Sequence Repeats, transposable elements (TE) were identified. In total 23 TEs were annotated, accounting for 7.4 kb of sequence and representing 4.6% of the BAC sequence (Table 2). The TE density reached one element per 6.97 kb. Transposable elements appeared uniformly distributed along the BAC sequence and no particular accumulation was observed. Only one putative element, weakly similar to the 3' part of a non-LTR retrotransposon in Arabidopsis (AAB82639), falls into the class I retrotransposon group. However, this element appears truncated and highly degenerated. Most annotated TEs belong to the class II transposon group. These identified elements are divided into non-autonomous transposons (five elements), and MITEs (Miniature inverted repeat transposable elements, 17 elements) representing respectively 21.7% and 73.9% of all identified elements in the BAC clone. One MITE, called *Alex-1 *was found nested within the 13th intron of an expressed gene encoding a putative protein (*g3*, position 12,468–12,645 bp, Figure 2).

**Table 2:** List of identified putative transposable elements in the *C. canephora *BAC 46C02.

**Class**	**Repeat Type**	**group**	**Number**	**Name**	***Coffea *EST BLAST hits (>10e-5)**	***Coffea *genomic sequence BLAST hits (>10e-5)**
**I**	Non-LTR retrotransposon	**LINE**	1	*CcRT_Pricilla*	0	0

**II**	Transposons	**Non-autonomous transposons**	5	*CcTR_Arnaud-1*	0	3
				*CcTR_Arnaud-2*	0	0
				*CcTR_Deless-1*	8	21
				*CcTR_Deless-2*	2	7
				*CcTR_Philippe*	7	2
		
	MITEs			*CcMT_Alex-1*	40	22
		!		*CcMT_Alex-2*	49	25
		!		*CcMT_Anis-1*	13	8
		!		*CcMT_Anis-2*	8	7
		!		*CcMT_Anis-3*	11	8
		!		*CcMT_Daniel-1*	17	6
		!		*CcMT_Daniel-2*	8	12
		!		*CcMT_Daniel-3*	15	15
		!		*CcMT_Gerard-1*	40	21
		!		*CcMT_Gerard-2*	37	14
		!		*CcMT_Jose-1*	1	8
		!		*CcMT_Jose-2*	4	7
		!		*CcMT_Jose-3*	0	0
		!		*CcMT_Jose-4*	0	1
		!		*CcMT_Richard*	1	0
		!		*CcMT_Serge*	1	9
		!		*CcMT_Stephane*	1	0

**Total**			23			

Similarity searches, using newly identified TEs as query, against publicly available *C*. ESTs and genomic sequences (to date 57,198 and 6,232 sequences, respectively from *C. canephora *and *C. arabica*) were conducted by BLAST with an E-value cutoff of 10e-5. Similarities were found for most of the identified elements (Table 2). Interestingly, two MITE families called *Alex *and *Gerard *were found as the most abundant in each sequence database, suggesting that these two elements belong to large copy number families in the *C. canephora *genome.

### Identifications of homologous regions between *C. canephora *BAC sequence and reference dicotyledonous genomes

Microcollinearity studies were conducted between the *C. canephora *BAC and the genomic sequences of five reference plants (tomato, grapevine, barrel medic, black cottonwood and Arabidopsis) belonging to Asterid and Rosid clades. Comparisons were achieved with completely and partially sequenced genomes.

**Figure 3:**
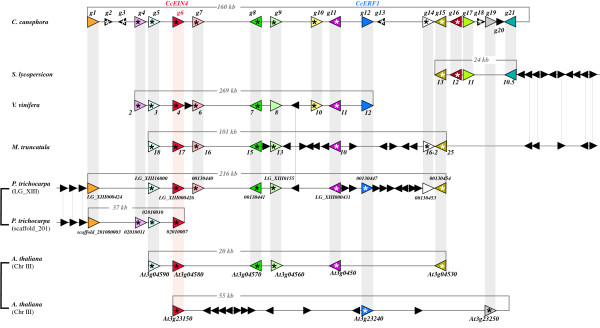
**Overview of the microcollinearity between *C. canephora *46C02 BAC and genomic regions in Arabidospsis (*Arabidopsis thaliana*), tomato (*S. lycopersicon*), Medigago (*Medicago truncatula*), grapevine (*V. vinifera*) and black cottonwood (*Populus trichocarpa*) genomes**. Colored arrows with names indicate orientation of predicted coding regions. Stars indicate transcribed genes as suggested by strong EST similarities (see Additional file 1). Colored lines link putative orthologous genes between collinear regions and distances between the most distant collinear genes with *C. canephora *BAC are indicated in each conserved fragment. Black arrows indicate non-conserved predicted genes. *S. lycopersicon *is a fragment (51–75 kb) of the Le_HBa0008H22 BAC clone located on chromosome 4. *V. vinifera *is a part of the scaffold127 of 1,150 kb long.*Medicago truncatula *indicates a fragment covered by two BAC clones (AC146861, AC173834). *Populus trichocarpa *corresponds to two fragments located in the linkage group XIII (3,073–3,289 kb) and in the unlinked scaffold 201 and *Arabidopsis thaliana *to two conserved fragments on chromosome III (A: 1,221–1,241 kb and B: 8,255–8,310 kb). Brackets link identify intra-genomic duplicated regions. The fragment sizes are not to scale.

The *C. canephora *BAC shows a partial conservation with a single BAC clone (Le_HBa0008H22 CU104691) located on the chromosome IV of tomato (*S. lycopersicum*, *Solanaceae*). Four *C. canephora *distal genes (*g15*, *g16*, *g17 *and *g21*), separated by an interval of 28 kb, were conserved in the same order and orientation in tomato on a distance of 24 kb (51,150–75,409 bp), giving a percentage of collinearity of 32%. In this interval, three *C. canephora *contiguous genes (*g18*, *g19 *and *g20*) were not found neither in the tomato counterpart nor in any other sequenced tomato BAC clones. Similarly, the other *C. canephora *genes were not found conserved in all released tomato BAC sequences (Figure 3).

The recently released grapevine genome (*Vitis vinifera*, Vitaceae [19]) allowed us to identify an additional conserved region with the *C. canephora *BAC sequence. In total nine *C. canephora *predicted genes (*g4*, *g5*, *CcEIN4*, *g7*, *g8*, *g9*, *g10*, *g11 *and *CcERF1*) were strictly conserved in the same order and orientation with the unmapped Scaffold127 of about 1,150 kb length (Le Cunff, L. and Adam-Blondon A.F., personal communication), representing a high percentage of collinearity (56%). Homologous grapevine genes spread on a large distance of 269.3 kb (675–945 kb), which represents an expansion of a factor 4.9 compared to the homologous counterpart in *C. canephora*. The increase of distances is primarily due to expansion of intergenic regions. Close examination and fine annotation of the grapevine 249 kb segment reveal the presence of numerous transposable elements in intergenic regions such as LTR retrotransposons and transposons (data not shown). Based on the identification of specific coding regions, the annotation showed the presence of nine complete and large size transposable elements. They account together for approximately 50 kb of sequence, indicating that the expansion of distance relative to the *C. canephora *segment may be due to a local accumulation of mobile elements in the grapevine counterpart.

The Cc*EIN4 *region shows a strong microcollinearity relationship with two overlapping barrel medic (*M. truncatula*; *Fabaceae*) BAC clones (AC146861 and AC173834) on chromosome 1 (Figure 3). In total, eight pairs of coding regions (*g5*, *CcEIN4*, *g7*, *g8*, *g9*, *g11*, *g14 *and *g15*) were found in the same order and orientation between scaffold 201 and Medicago, representing a percentage of collinearity of 40%. The Medicago conserved region spans 101 kb representing a limited contraction compared to the *C. canephora *conserved interval (107 kb), despite the identification of ten extra predicted genes in the Medicago interval compared to *C. canephora*. To date, the other *C. canephora *genes were not found by BLAST searches in Medicago sequences.

Comparisons between the *C. canephora *BAC and the complete black cottonwood genome (*P. trichocarpa*, Salicaceae) revealed extensive conservations with two different genomic fragments in populus, within a complex network of microcollinearity. Ten *C. canephora *coding regions (*g1*, *g5*, *CcEIN4*, *g7*, *g8*, *g9*, *g11*, *CcERF1*, *g14 *and *g15*) were first found in the same order and orientation with a fragment of 215.9 kb on chromosome XIII (positions on chr. XIII, 3,073–3,289 kb). In addition, four *C. canephora *coding regions (*g1*, *g4*, *g5 *and *CcEIN4*) were also conserved with a 37 kb part of the unanchored scaffold 201 (111–148 kb) (Figure 3). The percentage of collinearity is 49% and 32% for respectively the segment on chromosome XIII and the scaffold 201. Comparisons between *C. canephora *and black cottonwood homologous segments indicate a limited expansion of distance in black cottonwood compared to *C. canephora *of a factor 1,6 and 1 in chromosome XIII and scaffold 201 segments respectively (Figure 3).

Finally, comparisons between *C. canephora *and the *A. thaliana *(*Brassicaceae*) genome reveal a complex network of microcollinearity with two distinct regions located in the Arabidopsis chromosome III (Figure 3). Homologs of six *C. canephora *genes (*g5*, *CcEIN4*, *g8*, *g9*, *g11 *and *g15*) were conserved in the same order and orientation (collinearity of 44%) with a fragment 20 kb long on Arabidopsis Chromosome III at the position 1,221–1,241 kb. Here, four genes (*g7*, *g10*, *CcERF1 *and *g13*) on the *C. canephora *interval were not found conserved within the Arabidopsis homologous fragment. In *C. canephora*, conserved genes were separated by a distance of 107 kb, indicating an expansion of the fragment length by a factor 5.3 compared to Arabidopsis (Figure 3). Furthermore, three additional *C. canephora *genes (*CcEIN4*, *CcERF1 *and *g19*) were found conserved with an extra part of the Arabidopsis chromosome III. Here in the 55 kb of this later fragment (positions on Chr III 8,255–8,310 kb), eleven Arabidopsis genes were not found in the *C. canephora *sequence, that is indicated by the low collinearity percentage (17%). Compared to this second Arabidopsis homologous fragment, a 2.2 × expansion of the coffee segment length was observed. Analysis of the two Arabidopsis conserved fragments indicates an ancestral segmental duplicated block involving the two homologous Arabidopsis segments [20-22].

## Discussion

The high gene density in the *CcEIN4 *region represents a gene-rich segment of the *C. canephora *genome. The organization and composition of the trancriptionally active gene part of the *C. canephora *genome remain unexplored so far. At the *CcEIN4 *region, the gene density reaches one gene per 7.8 kb. Most of these genes (75%) were shown to be active or potentially active. This value is similar to the gene density observed in different euchromatic regions in tomato (genome size 950 Mb), one gene per 6.7 kb [23] and in the tomato *ovate *and *JOINTLESS *loci, one gene per 6.2 and per 8 kb, respectively [7,24]. Similarly, the gene density in the *C. canephora *46C02 BAC clone is comparable to euchromatic regions in *M. truncatula *(genome size ~500 Mb), one gene per 6.7 kb [25] and in rice, one gene per 6.7 kb [26]. However this density is relatively low compared to the Arabidopsis genome (125 Mb) by a factor ~2, one gene per 4 kb, [22,27], but still significantly high compared to the Populus genome (genome size 550 Mb), one gene per 11.1 kb, [28] and the euchromatic regions in sorghum (genome size 735 Mb), one gene per 12.3 kb, [29]. The *C. canephora *nuclei have been estimated to contain ~1.43 pg of DNA corresponding to a diploid genome, giving an estimated haploid genome size of ~690 Mb [10]. An extrapolation of the gene number identified in the 46C02 BAC clone to the whole *C. canephora *genome would lead to a predicted gene content of approximately 88,000 genes. This obvious over estimation is significantly higher than the average number of predicted genes in diploid reference plant genomes such as in tomato, ~38,000 [30], Arabidopsis, ~25,000 [22], grapevine ~30,000, Populus, 45,000 [28] and rice, ~37,000 [31]. Then, our data suggest that the high density observed at the *CcEIN4 *region may be not representative of the overall gene density of the *C. canephora *genome but to a high gene density zone. Gene enriched regions were previously identified in dicotyledonous plant genomes such as tomato and Medicago [23,32]. The tomato genome may cluster most of the active genes within 25% of the 950 Mb genome representing the euchromatin [23]. In Medicago, euchromatin, identified by FISH hybridization with gene-rich BAC clones, represents 20% of the 500 Mb genome [32]. These gene rich regions in the genomes of tomato and Medicago are currently targets of large-scale genome sequencing projects. Because genes are probably not uniformly distributed along *C. canephora *chromosomes, we hypothesize that the high gene density in the *CcEIN4 *region represents a gene-rich segment in the *C. canephora *genome.

The presence of a high number of identified MITEs in the *CcEIN4 *region is correlated with the presence of a high gene density. The transposable element composition of the BAC indicates a strong bias in the presence of Class II elements compared to Class I. With the exception of a degenerated part of a non-LTR retrotransposon, all identified elements have short lengths and are considered as non-autonomous because they lack coding capacities for a transposase involved in their mobility [33]. Due to their structural characteristics such as their small size (usually less than 500 bp), the presence of short Terminal Inverted Repeat (TIR) at both ends and a high A/T content, most of these elements (73.9%) were identified in *C. canephora *as Miniature Inverted-repeat Transposable Elements (MITE), a shorter derivative of non-autonomous class II DNA transposons [34,35]. MITE families were shown to be the most abundant TEs in plant genomes, with probably more than 90,000 copies in the rice genome [36]. Particularly, MITEs were frequently found associated with coding regions in plant genomic sequence with insertion into introns as well as into untranslated and promoter regions [24,37,38], which was confirmed with the complete sequencing of the Arabidopsis and rice genomes. Here, the identified MITEs were closely associated with coding regions as demonstrated by their distribution on the *C. canephora *BAC and their redundancies in *C. canephora *EST sequences.

The *CcEIN4 *region is located in the euchromatic part of the *C. canephora*. Although the organization of gene-rich regions along *C. canephora *chromosomes remains unknown, previous cytological observations in *C. canephora *and *C. arabica *chromosomes have clearly shown a pattern of deeply and lightly staining regions indicating an overall chromosome organization in respectively condensed heterochromatin and decondensed euchromatin regions [39,40]. Similarly to Medicago and tomato chromosome architecture, *C. canephora *heterochromatin regions are mainly located around centromeric regions while euchromatin constitutes the distal parts of chromosomes [13,32].

Based on these cytological observations and the similar composition observed between euchromatic regions in tomato, in Medicago and the sequenced BAC clone 46C02, we suggest that the *CcEIN4 *region is located in the euchromatic part of the *C. canephora *chromosome corresponding to the LG H of *C. heterocalyx*.

Comparison of the gene order between a *C. canephora *BAC clone and reference dicotyledonous genome sequences shows extensive conservation. Comparative genomic studies are essential approaches to understand the evolution of genome structure and to investigate the conservation of gene order between closely and distantly related plant species. The evaluation of the genome conservation allows the transfer of information from model species as references to "orphan" genomes lacking the availability of resources and may enhance the identification of gene of interest through map-based cloning strategies [41]. The sequencing of a gene rich region in *C. canephora *and the availability of several complete or forthcoming sequenced model dicotyledonous genomes such as Arabidopsis, black cottonwood, barrel medic, tomato and grape allowed us to investigate multiple microcollinearity relationships over distantly related species covering two different clades: Asterids and Rosids.

**Figure 4:**
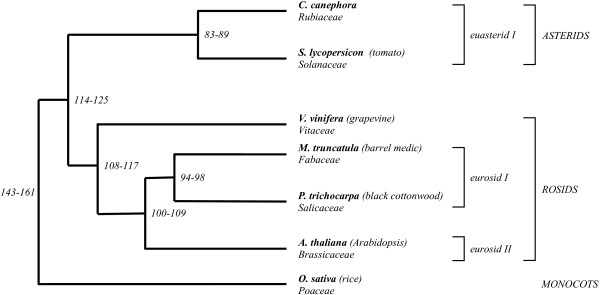
**Dendogram representing the phylogenetic relationships between *C. canephora *and reference eudicotyledonous genomes**. The time-scale of the divergence of the angiosperm families are indicated as published in Wilkstrom et al., [42].

The *CcEIN4 *region is clearly microcollinear with one homologous region in tomato, grapevine and Medicago and two homologous regions in Arabidopsis and in Populus genomes. Phylogenetically, *C. canephora *is closely related to tomato since they belong to the Asterid I clade and diverged from a common ancestor about 83–89 Million Years Ago (MYA) (Figure 4)[42]. Arabidopsis, Medicago, Populus and grapevine are more distant than tomato since they are all members of the Rosid clade, which diverged from the Asterid one 114–125 MYA (Figure 4). In the region studied here the number of collinear segments per genome and the degree of microcollinearity appeared heterogeneous. The *CcEIN4 *region is microcollinear with one homologous region both in tomato, grapevine and Medicago chromosomes, but scattered on two regions in Arabidopsis and in Populus genomes with a different degree of collinearity for each pair of homologous segments. These extra microcollinear segments lead to a complex network of conservation similar to that observed between tomato and Arabidopsis [6,7], Medicago and Arabidopsis [4,5], Medicago, soybean and Arabidopsis [43], Populus and Cucumis [9] and Arabidopsis and legumes genomes [5]. Such a network of conservation was recently suggested at synteny level between markers of the *C. arabica *S_H_3 region and Arabidopsis chromosomes 1, 3, 4 and 5 [44]. The analysis of networks of collinearity contributed to the discovery of recent large-scale chromosome duplications that shaped the genomes of Arabidopsis, Populus and Medicago, as well as the understanding of the evolutionary mechanisms that reorganized the duplicated blocks [20,21,28,45]. Moreover, recent comparative analysis between grapevine and the different reference genomes such as Arabidopsis, Populus and rice suggested no recent duplication, but an ancestral contribution of three genomes in grapevine common to all Eurosid plants [19], The data reported here confirm the paleopolyploid structure of the Arabidopsis and the Populus genomes. However, few collinear genes were found conserved between paralogous segments in Arabidopis and Populus, corresponding to the *C. canephora CcEIN4 *region. In Arabidopsis an extensive process of selective gene loss has shaped the paralogous segments while in Populus the duplicated block appeared very limited in length. Similar observations were described in other regions of paleopolyploid plant genomes where duplicated blocks were found subjected to intense mechanisms of gene movements and large rearrangements [20,28]. Although the presence of genome duplications and ancient triplication were respectively established in Medicago and suggested in grapevine, no network of collinearity was found between the *C. canephora CcEIN4 *region and these model species. Similar observations were found between the melon linkage group 11 and Medicago sequences [9] and between chickpea and Medicago sequences [46] reflecting the incomplete sequence of the Medicago genome or a region not covered by ancient polyploidization events in the Medicago and the grapevine genomes.

Considering the divergence between *C. canephora *and the model dicotyledonous plant genomes studied in this work, we expected a higher degree of microcollinearity between the *Rubiaceae *and the *Solanaceae *families than between *Asterid *and the distantly related *Rosid *clades. Synteny was observed between tomato and *C. canephora *in preliminary comparative mapping using COSII markers and microcollinearity relationships were previously demonstrated between tomato and the distant species Arabidopsis [6,7,24]. However, in the present work, only four genes were conserved in the same order and orientation over a similar distance and homologs of other *Coffea *genes were not present in all currently released tomato BAC sequences. Unfortunately, to date no tomato BAC sequence overlapping the entire homologous *C. canephora *BAC sequence is publicly available, suggesting that the homologous *CcEIN4 *region was not completely sequenced as part of the tomato genome project. Progress in the tomato sequencing project and future large scale genomic sequencing in *C. canephora *will allow us to re-evaluate the microcollinearity over several regions and to determine the limits of tomato genome as a tool to identify candidate genes in *C. canephora*. Nine genes were found strictly collinear and only two coding regions, located in the grapevine interval, were absent in the *C. canephora *counterpart. In contrast, an unexpected degree of microcollinearity was found between *C. canephora *and all Rosid plant genomes used in this study. The higher degree of microcollinearity (56%) corresponds to the comparisons between *C. canephora *and grapevine regions. In this later case, nine genes were found strictly collinear and only two coding regions, located in the grapevine interval, were absent in the *C. canephora *counterpart. Despite the high degree of microcollinearity, an expansion of distance of a factor 4.9 in grapevine was observed due to differential accumulations of transposable elements in intergenic spaces. This expansion of distance in grapevine is not correlated with the genome sizes in grapevine (~504 Mb, [47]) and in *C. canephora *(~690 Mb, [10]).

As it was previously observed in comparative analysis in grass genomes, massive insertions and deletions of TEs may promote a rapid genome evolution and may participate in the disruption of the microcollinearity [48]. Our analyses suggest that beside the alteration of distance, insertions of TE in grapevine show no impact on microcollinearity. Our observations indicate that a significant microcollinearity may be expected between *C. canephora *and grapevine. Phylogenetically, grapevine belongs to the *Vitaceae *family that is the earliest diverging lineage of the Rosid clade [49]. To date, it remains unclear whether the phylogenetic relationships of *Vitaceae *are at the origin of the local genome conservation with *C. canephora*.

The level of microcollinearity is still fairly high with Populus (LG XIII, 49%), Arabidopsis (segment A, 40%) and Medicago segments (40%) but lower than with grapevine. Several disruptions of the microcollinearity were observed between *C. canephora *and Populus, Arabidopsis and Medicago giving a mosaic pattern of conservation. In Populus and Medicago, the disruptions of the microcollinearity are due to the presence of numerous nonconserved genes with the *C. canephora *counterpart. Most of the extra genes in Populus and Medicago are not collinear across the Rosid model species suggesting that these gene insertions may arise from independent events of gene translocation mechanisms. Five genes located downstream the collinear region between Medicago and *C. canephora *were found to be collinear between Medicago and tomato. The extension of the microcollinearity pinpoints the complete absence in Medicago of a cluster of six genes present in *C. canephora *(*g16 *to *g21*), reinforcing the mosaic pattern of the gene conservation.

Despite the observation of the microcollinearity disruption, and considering the large number of collinear genes and distantly related species involved in this analysis, we conclude that the *C. canephora CcEIN4 *region is significantly conserved with all reference dicotyledonous species used in our analysis. Such observations raise the question whether the microcollinearity observed at the *CcEIN4 *region can be representative of the conservation between distantly related species or indicative of a larger conservation of the genome organization. So far, no other comparative analysis has been performed between *C. canephora *or any other Rubiaceae genomic sequences, and model dicotyledonous plant species. Further comparative mapping across dicotyledonous species will provide useful information about the conservation of the plant genome structure and also to determine a set of model genomes to infer positional information of candidate genes in *C. canephora*.

## Conclusion

In conclusion, our exploration of the first sequenced BAC clone in the *Rubiaceaae *family represents the first step in the understanding the *C. canephora *genome composition, structure, and evolution. Particularly *C. canephora *shows a remarkable level of microcollinearity with the distantly related Rosid species. In the absence of the complete tomato genome sequence, our data suggest the grapevine genome may be useful to obtain information to identify candidate genes in *C. canephora*.

## Methods

### Screening *C. canephora *BAC library and manipulation of BAC DNA

The screening of the *C. canephora *(clone IF 126) BAC library [12] representing approximately 9 genome equivalents was carried out using specific probe located in the previously isolated *CcEIN4 *gene [15]. The probe was amplified from the *CcEIN4 *cDNA with the following specific primers: SEIN4F 5'-GCCCTTGCGATTAATGAACCAG-3' and SEIN4R 5'-AGGCACAAGCACTTAACCAAACAA-3'. Probe preparation and BAC library high-density filters hybridization were performed as described in [16]. Through the hybridization, the BAC 46C02 was selected for further analysis. DNA of BAC 46C02 was isolated using the Plasmid Midi Kit (Qiagen Courtaboeuf, France) and used for further analyses. The insert size estimation was carried out by pulsed field (BioRad CHEF Gel Apparatus) with BAC DNA digested by *Not*I with the following parameters: volts/cm = 5.0; included angle 120°; run time = 15 hours at 14°C; initial switch time = 5 sec; final switch time = 15 sec.

### BAC-FISH mapping

The 46C02 BAC clone was labeled by random priming with biotin-14-dUTP (Invitrogen, Cergy-Pontoise, France). The ribosomal probe used in this study was pTa 71 [50] which contained a 9-kb *Eco*RI fragment of rDNA repeat unit (18S-5.8S-26S genes and spacers) isolated from *Triticum aestivum*. pTa 71 was labelled with Alexa-488 dUTP by random priming (Fisher Bioblock Scientific, Illkirch, France).

Chromosome preparations from *C. canephora *(genotype BB62, from Central African Republic) were incubated in Rnase A (100 ng/μL) and pepsin (0.05%) in 10 mmol HCL, fixed with paraformaldehyde (1%), dehydrated in an ethanol series (70%, 90% and 100%) and air-dried. The hybridization mixture consisted of 50% deionized formamide, 10% dextran sulfate, 2 × SSC, 1% SDS and labelled probes (200 ng per slide), was denatured at 92°C for 6 min, and transferred to ice. Chromosomes were denatured in a solution of 70% formamide in 2× SSC at 70°C for 2 min. The denatured probe was placed on the slide and *in situ *hybridization was carried out overnight in a moist chamber at 37°C. After hybridization, slides were washed for 5 min in 50% formamide in 2 × SSC at 42°C, followed by several washes in 4 × SSC-Tween. The chromosomes were mounted and counterstained in Vectashield (Vector Laboratories) containing 2.5 μg/mL 4',6-diamidino-2-phenylindole (DAPI). Fluorescence images were captured using a CoolSnap HQ camera (Photometrics, Tucson, Ariz) on an Axioplan 2 microscope (Zeiss, Oberkochen, Germany) and analysed using MetaVue™ (Universal Imaging Corporation, Downington, PA).

### BAC sequencing

The BAC DNA was first sub-cloned into the TOPO vector (Invitrogen, Cergy Pontoise, France) and 70 sub-clones were randomly selected for low-pass sequencing. Complete sequencing of the BAC 46C02 was performed by MWG Biotech (Ebersberg, Germany). A total of 1105 reads were produced giving an average coverage of 5.4×. After shotgun sequencing, sequences were assembled using the Phred/Phrap software [51], producing an assembly of 13 contigs. Regions of low quality as well as gaps between contigs were filled by PCR amplifications with specific primers and then sequenced (MWG Biotech). The final error rate for the BAC sequence was below 1 base per 10 kb. The BAC sequence was deposited in GenBank under the accession number EU164537.

### Genetic analysis

Two internal primers in the *CcEIN4 *gene were first designed (EIN4-RTF: AGAAGCTAGTTGGCATGTCCGGAT EIN4-RTR: GCAACTCGCAGCACCAAGTACTGA) and used as STS (Sequence Tag Site) for genetic mapping. The resulting marker was denominated EIN4. Using the SSR pipeline program [52], a microsatellite repeat [TC]_9 _(called BAC-37) was detected *in silico *at 105 kb away from the *CcEIN4 *gene. Flanking primers were designed (BAC-37-F: TCATTTTTGTCCGGGGATAC, BAC-37-R: ATGGAAACCGAAGAGGAAAG), and tested for amplification. PCR amplification was carried out on genomic DNA as described in [53]. Segregation analyses were performed on the interspecific progeny (74 individuals) derived from the back-cross [(*C. canephora *× *C. heterocalyx*) × *C. canephora*], which had been used to build a genetic map ([54] and unpublished results). Linkage analyses were performed using MAPMAKER/EXP 3.0b [55,56], and Mapdisto (version 1.37, available via ) software packages.

### Sequence Analysis and Gene Annotation Methods

The final BAC sequence was analyzed using BLAST algorithms [57] against public and local plant nucleotide and protein databases. Coding regions were first *ab initio *predicted using the FGENESH [58] gene finder software trained for three different eudicot gene species (*N. tabacum*, *S. lycopersicum *and *V. vinifera*) since no training set for *C. canephora *was available so far. Evaluation of predicted gene structures (i.e. coding regions, spliced sites, start and stop codons), were manually conducted by alignments with protein and nucleotide genomic sequences and confirmed by local alignments with *Coffea *public ESTs or specific cDNA PCR amplifications followed by sequencing. An additional genes structure validation was conducted by Felipe Rodrigues da Silva from the Cenergen (Embrapa) on *C. arabica *ESTs isolated by the Brazilian Coffee Genomic Consortium . Predicted genes with no similarity hits in all protein and nucleotide sequence databases were rejected in final annotation and considered as putative inaccurate predictions. Detailed analysis was performed with the EMBOSS Analysis software [59] and the final annotation was performed using Artemis [60].

### Annotation and classification of repeated sequences

Microsatellite (SSR) markers were identified using the SSR pipeline program with previously described parameters [52]. Putative transposable elements (TEs) were first identified and annotated by RepeatMasker  searches against local databases of nucleotide and protein sequences of known plant TEs. *De novo *prediction of TEs was performed according to the structure of the different class of TEs such as tandem and inverted repeats using dot-plot alignments, (Dotter software [61]). Putative TEs were named according to the following nomenclature: the first two letters of each TE name represent the acronym of the species (i.e. Cc for *Coffea canephora*), the following two letters indicate the type of TE (RT for retrotransposon; TR for transposon; MT for MITE, HL for helitron and UN for unknown class of repeat) and following a hyphen the specific name of the element.

### PCR Amplifications on cDNA libraries

Two *C. canephora *cDNA libraries, prepared from young leaves and fruits at different stages of development and maturation [62] were used for PCR amplifications with specific primers designed from genes identified on the BAC clone 46C02 (5'-GGCTGAGTTGGAACACTGGT-3' and 5'-TTAGGCTGGAAGCAAGAAGC-3' for 46C02_g1, 5'-GTTTGGTTGCTGGGTCTCAT-3' and 5'-CGACAAGAGGAAAGCCTCAC-3' for 46C02_g3, 5'-ACGAGTGGGTTTCCTGAGTG-3' and 5'-TGGGTCTCTGGAACTTACCG-3' for 46C02_g7, 5'-ACTCGGAGGCCTAGAGGAAG-3' and 5'-TAAAGCCATGACTGCACCAG-3' for 46C02_g8, 5'-GCTCTCAAACGTCCAAAACC-3' and 5'-AGCCTTTCCCACCTCTGTTT-3' 46C02_g9, 5'-GAAAACTTTCGCGCTCTTTG-3' and 5'-CCAGGTTGGATGTGCTTCTT-3' for 46C02_g10, 5' AATACCGCAATCTCGACACC-3' and 5'-ACGCAGTCCTATGCTCCTGT-3' for 46C02_g11, 5'-ATTCCCAGCATTGTCAGTCC-3' and 5'-TGCATCTGCTTCAACGACTC-3' for 46C02_g12, 5'-CTACTGCTTTGCTCGGGAAC-3' and 5'-GGAGCATGATCGTCTCCAAT-3' for 46C02_g13, 5'-GATGGAGAAATCCCAAATGC-3' and 5'-GACTGCAGGATGTTCAGCAA-3' for 46C02_g17, 5'-TCAAAAGTTTGAGTCGTTTGGA-3' and 5'-ACCAGCACTATCCCCACAAA-3' for 46C02_g18, 5'-GCAGGCTCATCTTTGCAAGT-3' and 5'-AAATGGGAAGGTTCATGCTG-3' for to 46C02_g20). Amplified products were directly sequenced without cloning.

### Analysis of the microcollinearity with plant genomes

To study in detail the microcollinearity relationships between *C. canephora *and model dicotyledonous genomes, the nucleotide and protein sequences of twenty predicted coding genes and one tRNA from the *C. canephora *BAC were used as queries for BLAST searches against a local database composed of *A. thaliana*, *S. lycopersicum*, *M. truncatula*, *P. trichocarpa *and *V. vinifera *nucleotide and protein sequences downloaded respectively from TAIR , SOL , *M. truncatula *sequencing resources , JGI  and the *V. vinifer*a genome database . Coding regions in non-annotated genomic sequences were identified using FGENESH [58] trained with the appropriate genome matrix. Collinear regions were defined as the conservation of a minimum of three genes in a maximum distance of 200 kb between compared sequences. In order to specify the robustness of the observed collinearity relationships we calculated a percentage of collinearity as the number of genes involved in the collinearity relationships in the two segments, divided by the total number of genes only present in the collinear part of the two segments compared.

## Authors' contributions

RG MdlM CC and JPB carried out the genomics and bioinformatics studies, VV, PH and OC performed FISH experiments, VP conducted the molecular genetic studies, RG and AdK managed the overall project and RG, AdK PH and SH contributed to the manuscript writing. All authors read and approved the final manuscript.

## Supplementary Material

Additional file 1 **List of homologous collinear predicted genes between *C. canephora *BAC 46C02 and Arabidospis, tomato, Medicago, grapevine and Populus**. The data provided represent all homologous *C. canephora *genes found by similarity searches and used to study the collinearity between *C. canephora *and sequenced genomes. Click here for file
