# Supplementary material for: Microcollinearity in an ethylene receptor coding gene region of the Coffea canephora genome is extensively conserved with Vitis vinifera and other distant dicotyledonous sequenced genomes
Source: BMC Plant Biol. 2009 Feb 25;9:22. doi: 10.1186/1471-2229-9-22 (PMC2656508; doi:10.1186/1471-2229-9-22)
Supplement: Additional file 1 — List of homologous collinear predicted genes between C. canephora BAC 46C02 and Arabidospis, tomato, Medicago, grapevine and Populus. The data provided represent all homologous C. canephora genes found by similarity searches and used to study the collinearity between C. canephora and sequenced genomes. [file 1471-2229-9-22-S1.pdf]

Additional files

Additional file 1. List of homologous collinear predicted genes between *C. canephora* BAC 46C02 and Arabidospis, tomato, Medicago, grapevine and Populus.

C. canephora genes	Homologous colinear genes	Organisms	TBLASTN e-value	ESTs	e-value	% of identity
g1	*Pt-fgenesh1_pm.C_scaffold_201000003*	*Populus trichocarpa*	e-123	/	/	/
g1	*Pt-fgenesh1_pg.C_LG_XIII000424*	*Populus trichocarpa*	e-123	/	/	/
g4	*Pt-eugene3.02010011*	*Populus trichocarpa*	e-27	DT475121	0.0	99%
g4	*Vv-scaffold_2*	*Vitis vinifera*	e-27	EC988821	e-120	100%
g5	*Vv-scaffold_3*	*Vitis vinifera*	e-56	EC940787	0.0	100%
g5	*At3g04590*	*Arabidopsis thaliana*	e-38	BX840094	0.0	99%
g5	*Pt-estExt_Genewise1_v1.C_LG_XIII1600*	*Populus trichocarpa*	e-51	CV255878	0.0	99%
g5	*Mt-AC173834_18*	*Medicago truncatula*	e-42	EY475600	0.0	100%
g5	*Pt-eugene3.02010010*	*Populus trichocarpa*	e-50	CK095843	0.0	97%
g6	*At3g04580*	*Arabidopsis thaliana*	0.0	AI993817	0.0	98%
g6	*Mt-AC173834_17*	*Medicago truncatula*	0.0	BQ255431	0.0	99%
g6	*Vv-scaffold_4*	*Vitis vinifera*	0.0	EC936812	0.0	99%
g6	*Pt-fgenesh1_pg.C_LG_XIII000426*	*Populus trichocarpa*	0.0	DN495148	0.0	99%
g6	*Pt-estExt_fgenesh1_pg_v1.C_2010007*	*Populus trichocarpa*	0.0	CV267968	0.0	99%
g6	*At3g23150*	*Arabidopsis thaliana*	0.0	DR368990	0.0	100%
g7	*Mt-AC173834_16*	*Medicago truncatula*	e-76	AL378861	0.0	100%
g7	*Vv-scaffold_6*	*Vitis vinifera*	e-59	EE106074	0.0	97%
g7	*Pt-eugene3.00130440*	*Populus trichocarpa*	e-113	DB888767	0.0	98%
g8	*Vv-scaffold_7*	*Vitis vinifera*	e-77	FC070242	0.0	100%
g8	*At3g04570*	*Arabidopsis thaliana*	e-48	DR325809	0.0	99%
g8	*Pt-eugene3.00130441*	*Populus trichocarpa*	e-71	DT515909	0.0	99%
g8	*Mt-AC173834_15*	*Medicago truncatula*	e-52	BE999313	0.0	99%
g9	*Vv-scaffold_8*	*Vitis vinifera*	e-101	BQ794023	0.0	100%
g9	*Mt-AC173834_13*	*Medicago truncatula*	e-107	CB895147	0.0	98%
g9	*At3g04560*	*Arabidopsis thaliana*	e-108	BX835785	0.0	99%
g9	*Pt-estExt_fgenesh1_pm_v1.C_LG_XIII0155*	*Populus trichocarpa*	e-117	CV280152	0.0	99%
g10	*Vv-scaffold_10*	*Vitis vinifera*	e-47	EC955073	0.0	100%
g11	*Pt-fgenesh1_pg.C_LG_XIII000431*	*Populus trichocarpa*	e-109	DT493292	0.0	99%
g11	*Mt-AC173834_10*	*Medicago truncatula*	e-99	EV256119	0.0	99%
g11	*At3g04550*	*Arabidopsis thaliana*	e-90	CK119472	0.0	99%
g11	*Vv-scaffold_11*	*Vitis vinifera*	e-115	CV093031	0.0	99%

g12	*Vv-scaffold_12*	*Vitis vinifera*	e-51	/	/	/
g12	*Pt-eugene3.00130447*	*Populus trichocarpa*	e-47	CV262586	0.0	99%
g12	*At3g23240*	*Arabidopsis thaliana*	e-44	DR751135	0.0	99%
g14	*Pt-eugene3.00130453*	*Populus trichocarpa*	e-177	/	/	/
g14	*Mt-AC146861_16*	*Medicago truncatula*	e-152	CX528565	0.0	100%
g15	*At3g04530*	*Arabidopsis thaliana*	e-91	CB254020	0.0	99%
g15	*Sl-CU104691_13*	*Solanum lycopersicon*	e-133	CK271232	0.0	96%
g15	*Pt-eugene3.00130454*	*Populus trichocarpa*	e-104	DB875035	0.0	99%
g15	*Mt-AC146861_25*	*Medicago truncatula*	e-106	EY477467	0.0	99%
g16	*Sl-CU104691_12*	*Solanum lycopersicon*	0.0	BP883946	0.0	100%
g16	*Pt-estExt_fgenesh1_pg_v1.C_LG_XIII0467*	*Populus trichocarpa*	0.0	CV260927	0.0	99%
g17	*Sl-CU104691_11*	*Solanum lycopersicon*	e-179	/	/	/
g19	*At3g23250*	*Arabidopsis thaliana*	e-58	DR750576	0.0	99%
g21	*Sl-CU104691_10.5*	*Solanum lycopersicon*	/	/	/	/
